# Axially Ligated Zirconium(IV) Tetraphenylporphyrin: Synthesis, Characterization, and Biological Activity

**DOI:** 10.1155/2014/543014

**Published:** 2014-10-14

**Authors:** Gauri D. Bajju, Sunil Kumar Anand, Gita Devi

**Affiliations:** P.G. Department of Chemistry, University of Jammu and Kashmir 180006, India

## Abstract

A series of 5,10,15,20-tetraphenylporphinatozirconium(IV) acetylacetonatophenolates containing different phenols as axial ligands [Zr(TPP)(Y)(X)] (TPP = 5,10.15,20-tetraphenyl-21H, 23H-porphine; Y = acac; X = different phenolates) have been synthesized and characterized by various spectrochemical studies. The complexes were also screened for antimicrobial activities. Antifungal activity of some adducts has been carried out against the fungal strain Sclerotium rolfsii. Most of the complexes have shown good antibacterial activity.

## 1. Introduction 

The present work is a continuation of our earlier work [1] where we have reported the synthesis of zirconium(IV) porphyrin complexes with salicylic acid and 5-sulfosalicylic acid which were made by replacing the two Cl^−^ by the organic ligands and this was followed by a biological study on some of these compounds and in view of the interesting results obtained from such axially substituted zirconium(IV) porphyrin it is considered worthwhile to make a study of axially substituted zirconium(IV) porphyrin with phenol and its derivatives. The ability of numerous chemical modifications and the large number of different mechanisms by which porphyrins affect microbial and viral pathogens place porphyrins into a group of compounds with an outstanding potential for discovery of novel agents, procedures, and materials active against pathogenic microorganisms [2]. A variety of biological activities exhibited by porphyrins are due to the fact that natural and synthetic porphyrins have relatively low toxicity* in vitro* and* in vivo* and they possess antitumor [3, 4] and antioxidant effects [4, 5] and have a good potential for metal ions complexation. Metalloporphyrins are the basis of new antifungal, antiparasitic, and anticancer drugs because modification of the porphyrin periphery confers qualitatively a new spectrum of activities to metalloporphyrins [6–8]. Zirconium(IV) porphyrins have gained attention from global researchers due to the peculiar characteristics of this class of compounds. The peculiarity of these complexes lies in the fact that metal ion in the complexes, that is, Zr^4+^, has large ionic radius (72 pm for most Zr(IV) 6-coordinate complexes), which fits partly into the core of porphyrin ligand and hence shows “out of plane” geometry with additional ligands always in cis position relative to the porphyrin plane [9]. The metal ion in these complexes is oxophilic [10] and thus may show preference for carboxylate and other oxygen-bearing anionic ligands. A lot of work is reported on the complexes of zirconium(IV) phthalocyanines [11–13] but comparatively less work has been done on zirconium(IV) porphyrin complexes with phenol as axial ligand [14, 15]. With this background in mind we reported herein the synthesis, spectroscopic characterization, and biological studies of a series of new axially substituted zirconium(IV) porphyrin with phenol and its derivatives as axial ligands.

## 2. Experiment

### 2.1. Materials and Instruments

All the chemicals were of analytical grade and used as received unless otherwise noted. Pyrrole was distilled over potassium hydroxide pellets under vacuum prior to use. All the organic solvents that were used for the synthesis and for chromatographic separations were dried before use. UV-vis spectra were recorded on a T90+ UV/VIS spectrophotometer in the range of 350–700 nm. The oscillator strength (*f*) of the transitions in absorption spectra was calculated from the expression

(1) $$\begin{array}{c} f=4.33\times 10^{-9}\varepsilon \Delta \nu_{1/2}, \end{array}$$

where *ε* is the molar absorption coefficient in dm^3^ mol^−1^ cm^−1^ and Δ*ν*_1/2_ is the full width at half maximum in cm^−1^. Infrared spectra were recorded on a PerkinElmer spectrum 400 FTIR spectrophotometer using KBr pellets in the range of 4000–400 cm^−1^. The elemental analysis was performed on Elemental Analyser CHNS-932, LECO, USA, at a temperature of about 1000°C using helium as carrier gas and oxygen for combustion. The ESI mass spectroscopy was recorded at room temperature and methanol was used as solvent. The ^1^H NMR spectra were recorded on a Bruker Avance II 500 (500 MHz) using tetramethylsilane as internal standard and CDCl_3_ as solvent. Fluorescence measurements were performed on Synergy MX BIOTEK multimode reader. The solution of porphyrins prepared in DMSO was 10^−6^ M.

### 2.2. Biological Studies

#### 2.2.1. Antibacterial Studies

Qualitative analysis for screening of antibacterial activity was carried out by agar-well diffusion method [16] with modifications. By measuring the inhibition zone in mm, the test compounds were taken at a concentration of 0.1 *μ*M using dimethyl sulfoxide (DMSO) as solvent. Chloramphenicol was used as positive control for antibacterial activity. The compound was tested against four gram positive bacteria (*Bacillus subtilis, Bacillus cereus, Staphylococcus aureus,* and* Enterococcus faecalis*) and four gram negative bacteria (*Klebsiella pneumonia, Alcaligenes denitrificans, Campylobacter,* and* Micrococcus luteus*). 20 mL of sterilized nutrient agar was inoculated with 100 *μ*L of bacterial suspension (10^8^ CFU/mL) and then poured on to sterilized Petri plate. The agar plate was left to solidify at room temperature. A well of 4 mm was aseptically bored into the agar plate. Then, 20 *μ*L of the complexes (diluted with DMSO) was added in each well. The plates were kept at 4°C for 2 hours to allow the dispersal and then incubated at 37°C for 24 hour.

#### 2.2.2. Antifungal Study

The antifungal activity of some adducts was tested against the pathogenic fungus* Sclerotium rolfsii* by poisoned food technique using potato dextrose agar (PDA) nutrient as the medium [17]. The linear growth of the fungus in controlled manner was recorded at different concentration of the adducts. The growth inhibition of* Sclerotium rolfsii* over control was calculated (Table 7). The growth inhibition of fungus over control was calculated as

(2) $$\begin{array}{c} \%\text{inhibition}\left(I\right)=\frac{C-T}{C}\times 100, \end{array}$$

where *I* is percent inhibition, *C* is mean growth of fungus (in mm) in control, and *T* is mean growth of fungus (in mm) in treatment.

### 2.3. Synthesis of Axially Ligated Zirconium(IV) Porphyrins Complexes

#### 2.3.1. Meso-5,10,15,20-tetraphenylporphyrin [H_2_TPP]

The H_2_TPP was prepared by refluxing benzaldehyde and pyrrole in propionic acid by following reported literature method [18] with modification.

#### 2.3.2. Synthesis of Axially Ligated Zr(IV) Porphyrins: [Zr(TPP)(Y)(X)]

**Scheme 1:**
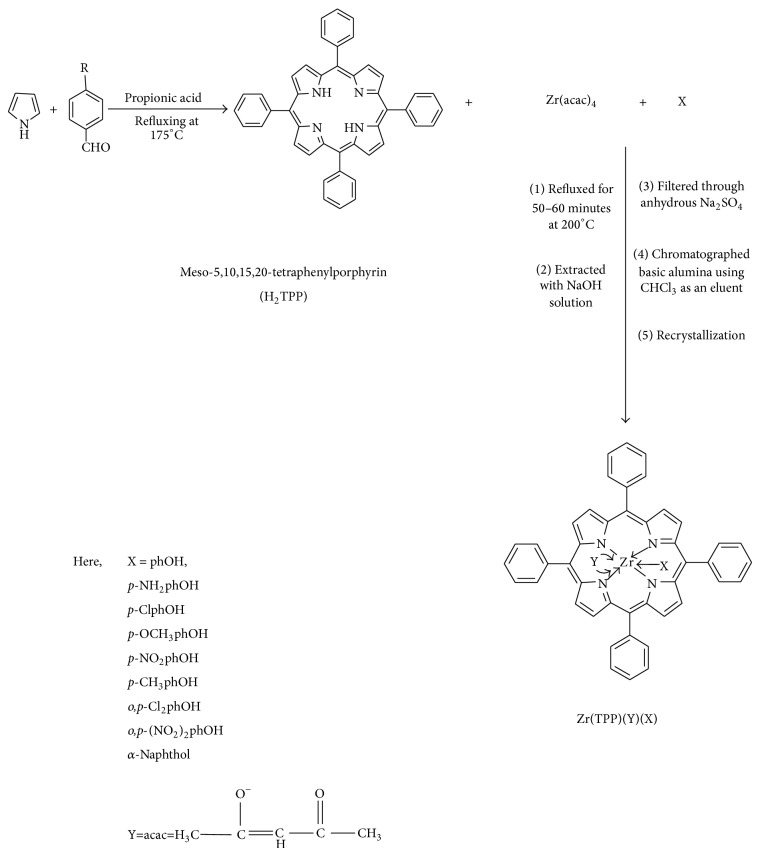
General synthetic route for the synthesis of axially ligated Zr(IV) porphyrins complexes.

A mixture of Zr(acac)_4_ (1.87 mmol), meso-tetraphenylporphyrin (3.74 mmol), and respective phenol (0.12 mmol) with constant stirring refluxed for about 50–60 minutes (Scheme 1). The reaction course was monitored by absorption spectra of the reaction mixture. After concentration, the mixture was dissolved in minimum quantity of CHCl_3_ and extracted with 2N NaOH solution to remove excess phenols. The lower layer containing compound in CHCl_3 _was collected and then it was filtered through anhydrous Na_2_SO_4_ in order to remove water and chromatographed through basic alumina using chloroform as an eluent and recrystallized from dichloromethane-hexane solution (1 : 1). The same procedure was applied for the synthesis of all axially ligated zirconium porphyrin complexes as described above. The purified axially ligated zirconium porphyrin complexes were obtained in yields of 35–40%.

*Zr(TPP)(acac)(Oph)*. Red solid; Anal. Calcd. for C_55_H_40_N_4_O_3_Zr: C 73.71, H 4.50, N 6.25; found: C 73.26, H 4.45, N 6.83; MS (CH_3_OH):* m/z* calcd. for C_55_H_40_N_4_O_3_Zr: 896.16; found 897.31 ([M+H]^+^); IR (KBr)*ν*_max⁡_: 473 cm^−1^ (*ν*_Zr–N_). 

*Zr(TPP)(acac)(p-NH*_*2*_ *phO)*. Yellow solid; Anal. Calcd. For C_55_H_41_N_5_O_3_Zr: C 72.50, H 4.54, N 7.69; found: C 72.62, H 4.52, N 7.62; MS (CH_3_OH):* m/z* calcd. for C_55_H_41_N_5_O_3_Zr: 911.17; found 912.01 ([M+H]^+^); IR (KBr)*ν*_max⁡_: 460 cm^−1^ (*ν*_Zr–N_). 

*Zr(TPP)(acac)(p-OCH*_*3*_ *phO)*. Red solid; Anal. Calcd. for C_56_H_42_N_4_O_4_Zr: C 72.62, H 4.57, N 6.05; found: C 71.23, H 4.96, N 5.62; ESI-MS (CH_3_OH):* m/z* calcd. for C_56_H_42_N_4_O_2_Zr: 926.18; found 927.34 ([M+H]^+^); IR (KBr)*ν*_max⁡_: 481 cm^−1^ (*ν*_Zr–N_).

*Zr(TPP)(acac)(p-CH*_*3*_ *phO)*. Red solid; Anal. Calcd. for C_56_H_42_N_4_O_3_Zr: C 73.90, H 4.65, N 6.16; found: C 73.65, H 4.88, N 5.42; ESI-MS (CH_3_OH):* m/z* calcd. for C_56_H_42_N_4_O_3_Zr: 910.18; found 911.25 ([M+H]^+^); IR (KBr)*ν*_max⁡_: 468 cm^−1^ (*ν*_Zr–N_).

*Zr(TPP)(acac)(p-ClphO)*. Brown solid; Anal. Calcd. for C_55_H_39_ClN_4_O_3_Zr: C 70.99, H 4.22, N 6.02; found: C 71.66, H 4.81, N 5.64; ESI-MS (CH_3_OH):* m/z* calcd. for C_55_H_39_ClN_4_O_3_Zr: 930.61; found 931.52 ([M+H]^+^); IR (KBr)*ν*_max⁡_: 475 cm^−1^ (*ν*_Zr–N_). 

*Zr(TPP)(acac)(p-NO*_*2*_ *phO)*. Brown solid; Anal. Calcd. for C_55_H_39_N_5_O_5_Zr: C 70.19, H 4.18, N 7.44; found: C 70.23, H 4.12, N 7.56; ESI-MS (CH_3_OH):* m/z* calcd. for C_51_H_32_N_4_O_3_Zr: 941.15; found 942.05 ([M+H]^+^); IR (KBr)*ν*_max⁡_: 484 cm^−1^ (*ν*_Zr–N_). 

*Zr(TPP)(acac)(o,p-Cl*_*2*_ *phO)*. Yellow solid; Anal. Calcd. for C_55_H_38_N_4_O_3_Cl_2_Zr: C 68.45, H 3.97, N 5.81; found: C 68.31, H 3.52, N 5.43; ESI-MS (CH_3_OH):* m/z* calcd. For C_55_H_38_N_4_O_3_Cl_2_Zr: 965.05; found 966.19 ([M+H]^+^); IR (KBr)*ν*_max⁡_: 480 cm^−1^ (*ν*_Zr–N_). 

*Zr(TPP)(acac)(o,p-(NO*_*2*_)_*2*_ *phO)*. Red solid; Anal. Calcd. for C_55_H_38_N_6_O_7_Zr: C 66.99, H 3.88, N 8.52; found: C 67.87, H 3.36, N 8.61; ESI-MS (CH_3_OH):* m/z* calcd. for C_55_H_38_N_6_O_7_Zr: 986.15; found 987.18 ([M+H]^+^); IR (KBr)*ν*_max⁡_: 478 cm^−1^ (*ν*_Zr–N_). 

*Zr(TPP)(acac)(*α*-naphtholate)*. Reddish brown solid; Anal. Calcd. for C_59_H_42_N_4_O_3_Zr: C 74.89, H 4.47, N 5.92; found: C 74.83, H 4.41, N 5.95; MS (CH_3_OH):* m/z* calcd. for C_59_H_42_N_4_O_3_Zr: 946.21; found 947.32 ([M+H]^+^); IR (KBr)*ν*_max⁡_: 473 cm^−1^ (*ν*_Zr–N_).

## 3. Results and Discussion 

### 3.1. Synthesis and Characterization

The general synthetic route to axially ligated zirconium(IV) porphyrins is shown in Scheme 1. All of these new zirconium(IV) porphyrins were purified by column chromatography with aluminum oxide as adsorbent and were characterized by spectral data (UV-visible spectroscopy, IR spectroscopy, ^1^H NMR spectroscopy, mass spectral data, and elemental analysis). The characterization data of the new compounds are consistent with the assigned formula. All the synthesized complexes are water insoluble.

#### 3.1.1. Spectral Analysis of Zr(TPP)(Y)(X)

**Figure 1:**
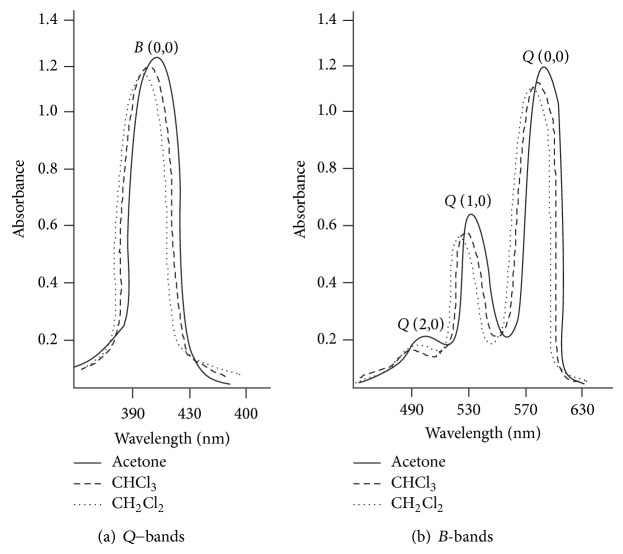
UV-vis spectra of Zr(TPP)(acac)(*p*-OCH_3_phO) in different solvent (—— Acetone, – – – CHCl_3_,…… CH_2_Cl_2_).

The spectral data of the synthesized complexes (Table 1) revealed that the axially ligated Zr(IV) metal derivatives of porphyrin with different phenolates as an axial ligand showed hypsochromic shift (blue shift) and variation in intensities of absorption bands when compared to their respective free base porphyrin, due to incorporation of the metal ion along with phenolate in the porphyrin rings [1, 19]. The complexes with electron donating groups in phenolates have slightly red shifted *B*- and *Q*-bands while those having electron withdrawing groups in phenolates have blue shifted *B* and *Q* bands. When the optical absorption spectra of the compounds of Zr(TPP)(Y)(X) were recorded in different solvents (Figure 1) only a marginal change in *λ*_max⁡_ values, absorption coefficient (*ε*), and oscillator strength (*f*) values was observed. Data revealed that a change in polarity of the solvent results in slight change in the position of transitions but there was a significant increase in *ν*_1/2_ and “*f*” values of transitions by increasing the polarity of the solvent (Table 2). The magnitude of change in “*f*” value in axially ligated Zr(IV) metal derivatives of porphyrin revealed the relative strength of *π*-*π* ^*^ interactions. It was also found that, with the increase in polarity of the solvents, *B* and *Q*-bands in axially ligated Zr(IV) metal derivatives showed red shift with progressive broadening of bands indicating that the magnitude of red shift of *B* and *Q* bands depends on the nature of the solvent used.

**Figure 2:**
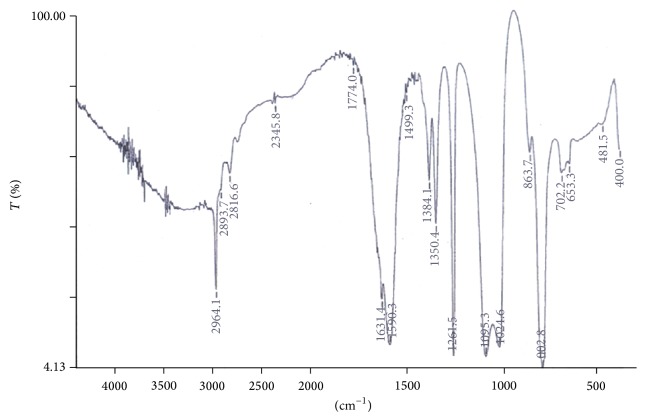
Infrared spectrum of Zr(TPP)(acac)(*p*-OCH_3_phO).

By comparing the infrared spectral data of H_2_TPP and its corresponding axially ligated Zr(TPP)(Y)(X) (Table 3), it is found that the band at 3447 cm^−1^ in H_2_TPP assigned to *ν*(N–H) (pyrrole) stretching vibration was disappeared in metallated complexes and the characteristic *ν*(Zr–N) vibration frequency found at ~500–430 cm^−1^, which indicated the formation of zirconium(IV) porphyrin compounds [20, 21]. In the spectra of all the axially ligated zirconium(IV) porphyrin complexes the incorporation of various phenolates in Zr(IV) metal derivatives of porphyrin, that is, Zr(TPP)(Y)(X), was confirmed by the appearance of Zr–O vibrational frequencies in the range of 649–680 cm^−1^indicating the coordination of phenolic oxygen to the metal via deprotonation (Figure 2). Also, the incorporation of acetylacetonate (acac) in axially ligated Zr(IV) derivatives was confirmed by the appearance of C=O vibrational frequencies in the range of 1622–1641 cm^−1^ and Zr–O in the range of 702–819 cm^−1^ corresponding to the ligation of zirconium to oxygen of phenolic and carboxylic groups, respectively [22, 23]. Thus, the zirconium atom in the centre of porphyrin ring coordinates with the acetylacetonate and phenol group axially to form seven-coordinate complex of Zr(IV) porphyrin.

From the^ 1^H NMR data of axially ligated zirconium(IV) porphyrin complexes in CDCl_3 _at 298 K (Table 4), it is found that the N–H protons of H_2_TPP appear at −2.77 ppm. In all the zirconium(IV) porphyrins there were absence of signal related to N–H protons and shift in other signals indicating the insertion of zirconium in porphyrin macrocycle [21]. Generally, the presence of Zr(IV) metal in the porphyrin ring shifts the resonances of the porphyrin's protons to downfield accompanied by marginal changes in the pattern. One of the important features of axially ligated Zr(IV) derivatives of porphyrins is that the metal is almost out of the plane of the porphyrin ring responsible for the production of asymmetric environment above and below the plane of the macrocycle which ultimately account for the pronounced no-equivalence of the orthoprotons of the phenyl rings.

The signals of axial phenol and acetylacetonate fragment protons are shifted to higher field in comparison to the signals of porphyrin protons and also in comparison to proton signals of free phenol and acetylacetonate, respectively. These positions of protons show that axial ligand is under the influence of *π*-conjugated system of porphyrin macrocycle [24]. The ^1^H NMR data of various axially ligated Zr(IV) compounds of H_2_TPP revealed that the presence of electron withdrawing groups like –NO_2_, –Cl at paraposition of phenolate caused slight downfield shift (deshielding) and the presence of electron releasing group like −CH_3_, −NH_2_ at paraposition of phenolate caused upfield shift (shielding) of protons with respect to Zr(TPP)(acac)(Oph) which have unsubstituted phenolate as an axial ligand. This is most probably due to deshielding effect resulting from the* σ*-donation of electron density upon bond formation as compared to the shielding effect of the porphyrin.

**Figure 3:**
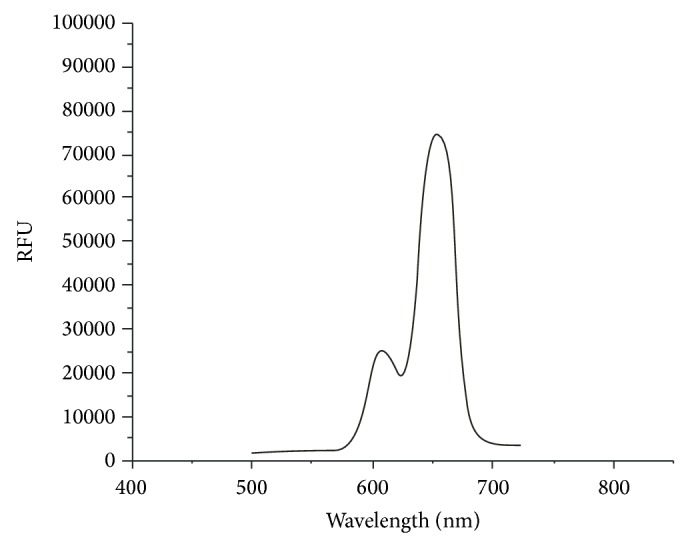
*S*_1_ → *S*_0_ fluorescence spectrum of Zr(TPP)(acac)(*p*-OCH_3_phO) in DMSO (*C* = 10^−6^ mol/L, *λ*_exc_ = 515 nm).

In the present investigation, the variation of emission properties in free base porphyrin H_2_TPP and some of its corresponding axially ligated Zr(IV) porphyrins has been studied (Table 5). The free base porphyrin exhibits two emission bands at 653 nm and 715 nm corresponding to *Q*(0,0) and *Q*(0,1) transitions, respectively, the intensity of the *Q*(0,0) being higher than the *Q*(0,1) transition. The axially ligated zirconium(IV) porphyrin complexes are emissive and show intraligand fluorescence comparable to other regular metalloporphyrins (Table 5). However, the emission bands of axially ligated Zr(IV) porphyrins are blue shifted compared to free base porphyrin (Figure 3). This behavior is attributed to an enhanced spin-orbit coupling induced by the presence of the heavy-atom central metals in zirconium(IV) porphyrins complexes, which leads to a more efficient intersystem crossing from the lowest porphyrin singlet excited state ^1^S_1_ (*π*, *π* ^*^) to the corresponding triplet manifold and thus reduces the probability of fluorescent emission [25]. Thus, the excitation spectrum of fluorescence is in agreement with absorption spectrum.

**Figure 4:**
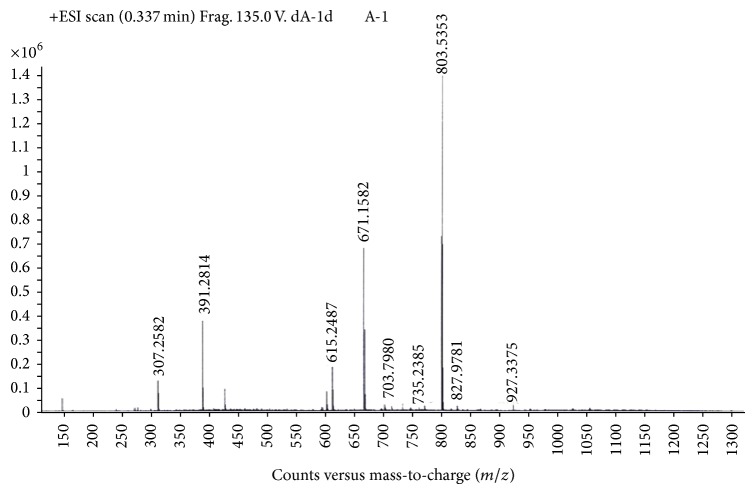
Mass spectrum of Zr(TPP)(acac)(*p*-OCH_3_phO) in methanol.

Mass spectrometric characterization of Zr(TPP)(Y)(X) complexes employed ESI as soft ionization technique. The mass spectra of axial ligated zirconium(IV) porphyrins are characterized by the presence of the molecular ion peak for monomeric form followed by a degree of fragmentation when employing this technique, which suggested that axial ligand was labile (Figure 4).

#### 3.1.2. Biological Studies

Antibacterial activity of all the synthesized zirconium(IV) porphyrin complexes was tested against eight bacterial strains, namely,* K. pneumonia, S. aureus, E. faecalis, A. denitrificans, B. cereus, M. luteus, B. subtilis, and Campylobacter* (Table 6). Our results demonstrated antibacterial activity against most of the zirconium(IV) porphyrin complexes and by comparing these complexes with H_2_TPP we noted that introducing zirconium and axial ligand in H_2_TPP increased antibacterial activity. Among all the complexes studied, Zr(TPP)(acac)(*p*-NO_2_phO) was found to be highly potential against all the eight bacterial strains with sensitivity ranging from 1 to 2.5 mm zone of inhibition and even more than positive control in some cases (Table 6). Zr(TPP) (acac)(*α*-naphtholate) was the only other complex after Zr(TPP)(acac)(*p*-NO_2_phO) complex that showed antibacterial sensitivity against all the bacterial strains with zone of inhibition ranging from 1 to 1.75 mm. On comparison of the antibacterial activities of synthesized complexes, we noted that for most of the bacterial strains complexes having axial ligand with electron withdrawing group have increased antibacterial activity compared to complexes having ligand with electron donating group and also compared to complex having no substituent on axial ligand, Zr(TPP)(acac)(phO).

#### 3.1.3. Antifungal Activity

The antifungal activity of all the synthesized zirconium porphyrin complexes was tested at different concentrations against the pathogenic fungus* Sclerotium rolfsii.* From the results found, it has been concluded that, by increasing the concentration of the complexes ZrTPP(Y)(X), the colony diameter of the fungus decreases and hence percent inhibition increases. On doubling the concentration of the complexes, the percent inhibition also doubles, which shows linear relationship between concentration and percent inhibition. The increase in antimicrobial activity is due to faster diffusion of metal complexes as a whole through the cell membrane or due to combined activity effect of the metal and the ligand [26, 27]. It is concluded that most of the synthesized compounds showed overall good activity. However, some complexes, namely, Zr(TPP)(acac)(phO), Zr(TPP)(acac)(*o,p*-Cl_2_phO), and Zr(TPP)(acac)(*o,p*-(NO_2_)_2_phO), showed negligible results at given concentrations and the data for only those complexes has been provided which showed significant results (Table 7).

It is interesting to note that most of the synthesized axially ligated complexes were found to be more active (IC_50_ = ~26–196 *μ*g/mL) than the corresponding free base ligand (IC_50_= 212.24 *μ*g/mL) with Zr(TPP)(acac)(*p*-CH_3_phO) appearing to be the most potent. The selectivity might be resulting from the well-established structural differences between fungal and bacterial cells, although the exact reasons remain as yet unclear [28].

## 4. Conclusion

A detailed analysis of ultraviolet-visible (UV-vis), proton nuclear magnetic resonance (^1^H NMR) spectroscopy, infrared (IR) spectroscopy, fluorescence and mass spectroscopic studies, and elemental analysis suggested the transformation from free base porphyrins to zirconium(IV) porphyrins. The spectroscopic data revealed the ligation of acetylacetonate and different phenolates at axial position on Zr(IV) metal atom in [Zr(TPP)(acac)(X)]. Therefore the coordination number of central metal ions is seven and the zirconium is expected to be above the porphyrin plane. Among all the complexes prepared [Zr(TPP)(acac)(*p*-NO_2_phO)] was found to be highly potential against all the eight bacterial strains and even more than positive control in some cases. Also, antifungal activity of the synthesized complexes shows that these complexes have potential against fungal growth.

## Figures and Tables

**Table 1:** Optical absorption data of Zr(TPP)(Y)(X) complexes in CHCl_3_.

Compounds	*B*-bands *λ*_max⁡_ (nm), (log⁡*ε*)	*Q*-bands *λ*_max⁡_(nm), (log⁡*ε*)
Zr(TPP)(acac)(Oph)	413.1, (5.074)	500.5, (4.183) 535.7, (4.796) 579.9, (5.009)

Zr(TPP)(acac)(*p*-OCH_3_phO)	414.2, (5.101)	501.5, (4.197) 536.8, (4.812) 580.9, (5.031)

Zr(TPP)(acac)(*p*-CH_3_phO)	413.9, (5.104)	501.7, (4.200) 537.4, (4.814) 581.4, (5.033)

Zr(TPP)(acac)(*p*-NO_2_phO)	412.9, (5.056)	501.4, (4.168) 535.1, (4.788) 580.8, (4.990)

Zr(TPP)(acac)(*p*-ClphO)	413.3, (5.068)	500.8, (4.177) 536.1, (4.795) 580.2, (5.005)

Zr(TPP)(acac)(*p*-NH_2_phO)	415.1, (5.119)	502.9, (4.214) 538.9, (4.828) 583.5, (5.049)

Zr(TPP)(acac)(*o,p*-Cl_2_phO)	411.8, (5.061)	499.2, (4.172) 534.3, (4.793) 578.8, (4.997)

Zr(TPP)(acac)(*o,p*-(NO_2_)_2_phO)	410.9, (5.049)	498.5, (4.159) 532.9, (4.781) 578.3, (4.472)

Zr(TPP)(acac)(*α*-naphtholate)	412.8, (5.068)	500.3, (4.178) 535.4, (4.795) 579.7, (5.007)

**Table 2:** Optical absorption data of Zr(TPP)(Y)(X) in different solvents.

Compounds	Solvent	*λ*_max⁡_ (nm), log⁡*ε* (M^−1^ cm^−1^)				*ν*_1/2_ (cm^−1^)		*Q*(0, 0) *f*
		*B*(0,0)	*Q*(2,0)	*Q*(1,0)	*Q*(0,0)	*B*(0,0)	*Q*(0,0)	
Zr(TPP)(acac)(phO)	Acetone	413.1	500.5	535.7	579.9	1309.3	1067.2	0.235887
		5.074	4.183	4.796	5.009			
	CH_2_Cl_2_	409.4	496.3	532.2	575.8	1241.6	1020.7	0.197404
		5.030	4.126	4.721	4.951			
	CHCl_3_	411.3	498.5	533.4	577.5	1278.1	1041.1	0.207468
		5.042	4.139	4.735	4.964			

Zr(TPP)(acac)(*p-*OCH_3_phO)	Acetone	414.2	501.5	536.8	580.9	1340.2	1085.9	0.252492
		5.101	4.197	4.812	5.031			
	CH_2_Cl_2_	409.8	496.4	530.6	574.9	1279.9	1039.3	0.207586
		5.057	4.172	4.749	4.965			
	CHCl_3_	411.9	498.3	534.2	578.5	1312.6	1062.4	0.213671
		5.063	4.178	4.765	4.968			

Zr(TPP)(acac)(*p*-CH_3_phO)	Acetone	412.9	501.4	535.1	580.8	1270.3	1035.2	0.219019
		5.056	4.168	4.788	4.990			
	CH_2_Cl_2_	409.7	496.3	532.3	576.4	1218.3	992.6	0.183752
		5.002	4.131	4.718	4.932			
	CHCl_3_	411.2	499.1	533.8	577.9	1236.8	1007.9	0.194033
		5.012	4.147	4.729	4.949			

Zr(TPP)(acac)(*p*-NO_2_phO)	Acetone	410.9	500.2	536.1	582.5	1271.2	1033.2	0.256245
		5.050	4.156	4.728	4.901			
	CH_2_Cl_2_	408.6	495.2	531.5	576.3	1218.1	991.3	0.191456
		5.010	4.111	4.513	4.899			
	CHCl_3_	410.1	498.9	537.6	577.7	1235.9	1006.7	0.198565
		5.006	4.124	4.279	4.999			

Zr(TPP)(acac)(*α*-naphtholate)	Acetone	413.5	500.4	535.2	581.9	1272.3	1034.2	0.232365
		5.045	4.116	4.718	4.912			
	CH_2_Cl_2_	411.5	497.8	535.6	577.6	1217.3	9956	0.182536
		5.013	4.113	4.518	4.922			
	CHCl_3_	413.5	498.3	535.8	578.9	1235.8	1005.3	0.195632
		5.102	4.127	4.739	4.948			

**Table 3:** Main vibrational frequencies of axially ligated Zr(IV) porphyrin complexes.

Porphyrin	*ν*(N–H) (cm^−1^)	*ν*(C=C) (cm^−1^)	*ν*(Zr–N) (cm^−1^)	*ν*(Zr–O) phenolate (cm^−1^)	*ν*(CH_3_) (cm^−1^)	*ν*(OCH_3_) (cm^−1^)	*ν*(NH_2_) (cm^−1^)	*ν*(NO_2_) (cm^−1^)	*ν*(C–Cl) (cm^−1^)	*ν*(Zr–O) acac (cm^−1^)	*ν*(C=O) acac (cm^−1^)
Zr(TPP)(acac)(Oph)	—	1590	473	664	2907	—	—	—	—	703 803	1622

Zr(TPP)(acac)(*p*-NH_2_phO)	—	1592	460	655	2891	—	*ν*(NH_2_)_sym_ = 3292 *ν*(NH_2_)_asym_ = 3366	—	—	712 804	1623

Zr(TPP)(acac)(*p*-OCH_3_phO)	—	1590	481	653	2894	*ν*(C–H) = 2817 *ν*(COC)_sym_ = 1025 *ν*(COC)_asym_ = 1261	—	—	—	702 802	1631

Zr(TPP)(acac)(*p*-CH_3_phO)	—	1584	468	651	2895	—	—	—	—	702 803	1630

Zr(TPP)(acac)(*p*-ClphO)	—	1591	475	666	2906	—	—	—	783	704 813	1634

Zr(TPP)(acac)(*p*-NO_2_phO)	—	1592	484	667	2909	—	—	1342 1543	—	713 810	1636

Zr(TPP)(acac)(*o,p*-Cl_2_phO)	—	1595	480	669	2907	—	—	—	788	716 804	1625

Zr(TPP)(acac)(*o,p*-(NO_2_)_2_phO)	—	1596	478	668	2905	—	—	—	789	705 804	1639

Zr(TPP)(acac)(*α*-naphtholate)	—	1589	473	659	2897	—	—	—	—	709 806	1632

**Table 4:** ^
1^H NMR data of Zr(TPP)(Y)(X) in CDCl_3_.

Porphyrins	Imino protons	*β*-Pyrrole protons	Meso-aryl protons	acac protons	Phenolate protons
Zr(TPP)(acac)(phO)	—	8.94 (s)	8.26 (d, 4H, Ho) 7.79 (d, 4H, Ho) 7.68–7.79 (m, 12H, Hm, p)	1.46 (s, 6H, H_CH_3__) 4.56 (s, H, H_CH_)	7.04 (d, 2H, Ho) 7.14–7.27 (m, 3H, Hm, p)

Zr(TPP)(acac)(*p*-NH_2_phO)	—	8.42 (s)	7.45 (d, 4H, Ho) 7.28 (d, 4H, Ho) 7.11–7.19 (m, 12H, Hm, p)	1.52 (s, 6H, H_CH_3__) 3.85 (s, H, H_CH_)	6.87 (d, 2H, Ho) 6.71 (d, 2H, Hm) 4.85 (s, 2H, H_NH_)

Zr(TPP)(acac)(*p*-OCH_3_phO)	—	8.47 (s)	7.50 (d, 4H, Ho) 7.40 (d, 4H, Ho) 7.16–7.24 (m, 12H, Hm, p)	1.55 (s, 6H, H_CH_3__) 3.89 (s, H, H_CH_)	6.98 (m, 4H, Ho, m) 3.43 (s, 3H, H_OCH_3__)

Zr(TPP)(acac)(*p*-CH_3_phO)	—	8.47 (s)	7.48 (d, 4H, Ho) 7.74 (d, 4H, Ho) 7.15–7.23 (m, 12H, Hm, p)	1.50 (s, 6H, H_CH_3__) 3.89 (s, H, H_CH_)	6.98 (m, 4H, Ho, m) 2.18 (s, 3H, H_CH_3__)

Zr(TPP)(acac)(*p*-ClphO)	—	9.34 (s)	8.37 (d, 4H, Ho) 8.18 (d, 4H, Ho) 7.83–7.92 (m, 12H, Hm, p)	1.79 (s, 6H, H_CH_3__) 4.59 (s, H, H_CH_)	7.12 (d, 2H, Ho) 7.36 (d, 2H, Hm)

Zr(TPP)(acac)(*p*-NO_2_phO)	—	9.36 (s)	8.49 (d, 4H, Ho) 8.21 (d, 4H, Ho) 7.88–7.97 (m, 12H, Hm, p)	1.81 (s, 6H, H_CH_3__) 4.63 (s, H, H_CH_)	7.21 (d, 2H, Ho) 7.42 (d, 2H, Hm)

Zr(TPP)(acac)(*o,p*-Cl_2_phO)	—	8.51 (s)	8.48 (d, 4H, Ho) 8.21 (d, 4H, Ho) 7.95–8.05 (m, 12H, Hm, p)	1.80 (s, 6H, H_CH_3__) 4.64 (s, H, H_CH_)	7.12 (s, 1H, Ho) 7.66–7.82 (m, 2H, Hm)

Zr(TPP)(acac)(*o,p*-(NO_2_)_2_phO)	—	9.57 (s)	8.57 (d, 4H, Ho) 8.29 (d, 4H, Ho) 8.05–8.13 (m, 12H, Hm, p)	2.11 (s, 6H, H_CH_3__) 4.72 (s, H, H_CH_)	7.22 (s, 1H, Ho) 7.72–7.86 (m, 2H, Hm)

**Table 5:** Summary of the fluorescence band maxima at 23 K in DMSO.

Compound	*λ*_max⁡_, nm		
	*B*(0,0)	*Q*(0,0)	*Q*(0,1)
H_2_TPP	450	653	715
Zr(TPP)(acac)(*p*-OCH_3_phO)	440	609	660
Zr(TPP)(acac)(*p-*CH_3_phO)	440	608	657
Zr(TPP)(acac)(*p*-NO_2_phO)	443	610	663
Zr(TPP)(acac)(*α*-naphtholate)	441	608	653

**Table 6:** *In vitro* antibacterial evaluation of free base porphyrin and the corresponding zirconium(IV) porphyrin complexes.

PORPHYRIN	*K. pneumoniae *	*S. aureus *	*E. faecalis *	*A. denitrificans *	*B. cereus *	*M. luteus *	*B. subtilis *	*Campylobacter *
H_2_TPP	—	—	1	—	—	—	—	—
Zr(TPP)(acac)(phO)	—	—	1.25	—	—	—	—	—
Zr(TPP)(acac)(*p*-NH_2_phO)	—	—	—	1.5	—	—	—	—
Zr(TPP)(acac)(*p*-ClphO)	—	—	—	—	—	—	—	1.5
Zr(TPP)(acac)(*p*-OCH_3_phO)	—	—	—	1	—	—	—	—
Zr(TPP)(acac)(*α*-naphtholate)	1	1.15	1.1	1.25	1.1	1.5	1.75	1
Zr(TPP)(acac)(*p*-NO_2_phO)	2	1	1.5	1.5	1.2	1.4	2.5	1.5
Zr(TPP)(acac)(*p*-CH_3_phO)	1	0.7	1.7	—	0.9	—	0.7	0.7
Zr(TPP)(acac)(*o,p*-Cl_2_phO)	—	—	—	0.9	—	—	—	1.25
Zr(TPP)(acac)(*o,p*-(NO_2_)_2_phO)	—	1.25	1.25	—	—	2	—	1.5
Control chloramphenicol	2.5	2.1	1.4	2	—	2.25	2	2

**Table 7:** *In vitro* evaluation of complexes against *Sclerotium rolfsii*. Mean colony diameter of control *C* = 90 mm.

Name of the complex	Concentration (*µ*g/mL)	Colony diameter (mm)	% Inhibition *I* = [(*C* − *T*)/*C*] × 100	IC_50_ (*µ*g/mL)
H_2_TPP	100	66	26.66	212.24
	200	48	46.66	
	300	28	68.89	

Zr(TPP)(acac)(*p*-NH_2_phO)	100	57	36.66	180.61
	200	39	56.66	
	300	11	87.77	

Zr(TPP)(acac)(*p*-ClphO)	100	61	32.22	159.42
	200	43	52.22	
	300	12	86.66	

Zr(TPP)(acac)(*p*-OCH_3_phO)	100	65	27.77	196.46
	200	41	54.44	
	300	27	70	

Zr(TPP)(acac)(*α*-naphtholate)	100	33	53.33	102.75
	200	30	66.66	
	300	8	91.11	

Zr(TPP)(acac)(*p*-NO_2_phO)	100	35	51.11	91.87
	200	23	73.33	
	300	7	92.22	

Zr(TPP)(acac)(*p*-CH_3_phO)	100	35	61.11	26.02
	200	21	77.77	
	300	7	92.22	
